# Zeta Potentials of Magnetite Particles and Alloy 690 Surfaces in Alkaline Solutions

**DOI:** 10.3390/ma13183999

**Published:** 2020-09-09

**Authors:** Ji-Min Lee, Dong-Seok Lim, Soon-Hyeok Jeon, Do Haeng Hur

**Affiliations:** 1 Materials Safety Technology Development Division, Korea Atomic Energy Research Institute, 989-111, Deadeok-Daero, Yuseong-gu, Daejeon 34057, Korea; jmlee0915@kaeri.re.kr (J.-M.L.); limds@kaeri.re.kr (D.-S.L.); junsoon@kaeri.re.kr (S.-H.J.); 2 Department of Materials Science and Engineering, Chungnam National University, 99, Daehak-ro, Yuseong-gu, Daejeon 34134, Korea

**Keywords:** zeta potential, magnetite, Alloy 690, steam generator, water chemistry, pH, pH agent, NaCl

## Abstract

Magnetite particles deposited on the secondary side of a steam generator (SG) can degrade the integrity and performance of pressurized water reactors. Therefore, it is necessary to produce the data of fundamental interfacial electrokinetic properties of magnetite particles and SG tube materials. This study investigated the zeta potentials of magnetite nanoparticles and Alloy 690 surfaces, which were dependent on the pH value, pH agent, and the presence of NaCl. The zeta potentials of the magnetite nanoparticles increased in the negative direction as the pH increased, regardless of the pH agent. At the same pH value, the absolute values of the zeta potentials with different pH agents were: ethanolamine < ammonia < morpholine. In the presence of NaCl, the zeta potentials of the particles further increased negatively. The meaning of the measured zeta potentials was discussed in terms of the dispersion stability and the agglomeration of the particles. Based on the relationship between the zeta potentials of the particles and Alloy 690 surfaces, the magnetite deposition on Alloy 690 was also discussed. Furthermore, the empirical formulas for the pH-dependent zeta potentials of magnetite particles in each alkaline solution were suggested.

## 1. Introduction

Corrosion products of carbon steel and other structural materials in pressurized water reactors (PWRs) are released to the secondary water as a result of flow-accelerated corrosion [1,2]. These corrosion product particles are transported to steam generators (SGs) and attached to SG tubes to form deposits with a porous structure. The majority of these deposits are composed of magnetite [3]. These magnetite deposits reduce the heat transfer efficiency, hinder the normal water flow, accelerate the corrosion of the SG tube materials owing to the galvanic effect, and even act as a site where aggressive impurities can accumulate [4,5,6]. Therefore, mitigating and inhibiting such magnetite deposition is a key to controlling the chemistry of secondary water for reliable PWR operation.

To achieve this goal, it is essential to produce fundamental dispersion property data of magnetite particles suspended in secondary water. The degree of dispersion stability of colloidal particles in an aqueous solution can be evaluated by determining the zeta potential, which is the principal electrokinetic property of the particles and governs their agglomeration, dispersion, and deposition on metal surfaces [7,8]. The degree of zeta potential is typically measured in mV and determines the degree of electrostatic repulsion between adjacent particles. For example, as the zeta potential approaches zero, the particles tend to agglomerate; however, the repulsive force of each particle increases as the zeta potential increases, thereby increasing the dispersion stability in the solution. On the other hand, like particles in suspension, any metal surface in an aqueous environment can acquire a zeta potential. Accordingly, the metal surface can interact with the adjacent charged particles. Recent studies have demonstrated that the number of particles deposited on a metal surface is strongly affected by the difference in the zeta potentials of the particles and metal surfaces [9,10]. Therefore, comparing the zeta potentials of magnetite particles and SG tube materials could prove useful for studying magnetite deposition behavior in the secondary side of SGs.

The secondary side of PWRs is managed by a thorough pH control to suppress the corrosion of the structural materials of the secondary system. In general, the pH has been controlled to approximately 9.0–10.0 with ammonia, morpholine, or ethanolamine (ETA), which are the most widely used pH control agents. Although the zeta potential data of magnetite particles in the PWRs are critical, numerous previous studies have only reported the zeta potentials of magnetite particles in alkaline solutions using NaOH or KOH, which are not used in the nuclear industry. In addition, NaCl, which can be introduced to secondary water by condenser in-leakage, can cause stress corrosion cracking or pitting corrosion of SG tubes [11,12]; therefore, its concentration has been strictly regulated less than 5 ppb for Na and 10 ppb for Cl, based on the water chemistry guideline values during SG operation [13,14]. However, few studies have been conducted on how NaCl affects the zeta potential of magnetite particles in nuclear water chemistry. Furthermore, a literature survey showed that there have been few studies on the surface zeta potential of SG tube materials in secondary water.

Thus, in this study, when the pH was controlled with ammonia, morpholine, or ETA, the pH-dependent zeta potentials of magnetite nanoparticles were measured and compared. The effect of the addition of NaCl on the zeta potential of magnetite nanoparticles was also investigated. Accordingly, the meaning of the measured zeta potentials was qualitatively discussed in terms of the dispersion stability and the agglomeration of the particles. Additionally, the surface zeta potentials of Ni-based thermally treated Alloy 690 were measured, which is the primary SG tube material used in PWRs because of its excellent corrosion resistance. Subsequently, the magnetite deposition on Alloy 690 was discussed by comparing the measured zeta potentials of magnetite particles and Alloy 690 surfaces.

## 2. Experimental

### 2.1. Zeta Potential Measurement

**Figure 1:**
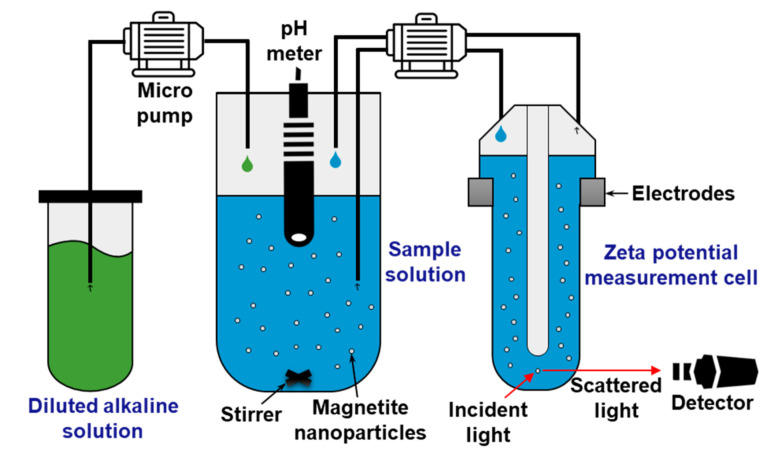
Schematic of zeta potential measurement setup using an automatic pH titration system.

Figure 1 shows a schematic of the zeta potential measurement using a Zetasizer (Nano ZS90) equipped with an automatic pH-titrator (MPT-2) (Malvern Panalytical Ltd., Worcestershire, UK). Initially, we prepared a sample solution by dispersing magnetite nanoparticles in deionized water at a concentration of 25 mg/L. The magnetite nanoparticles were purchased from Sigma-Aldrich Co. (St. Louis, MO, USA), whose size ranged from 4 nm to 6 nm with an average of 5 nm. Additionally, a diluted alkaline solution of ammonia, morpholine, or ETA was prepared. The basic properties of these three pH agents used in this study are presented in Table 1. Subsequently, the alkaline solution was injected into the sample solution using a programmable micrometering pump, and the pH of the sample was titrated in real-time. Afterwards, when the pH reached the target value, the sample was automatically transferred to the zeta potential measurement cell. After the stabilization time, the electric field was applied through an electrode pair mounted on both sides of the cell. The charged magnetite particles were then attracted toward the oppositely charged electrode with a velocity proportional to the field strength and charge. The electrophoretic mobility of the particles was measured by the electrophoretic light scattering (ELS) technique that is known as the most suitable method for determining the zeta potential of particles fine enough to stay suspended in a solution [7]. The measured mobility was then converted to the zeta potential (ζ) through Henry’s equation [15] expressed as:

(1) $$\zeta =\frac{\eta U_{E}}{\varepsilon_{r}\varepsilon_{0}f\left(\kappa a\right)}$$

(2) $$f\left(\kappa a\right)=\frac{2}{3}\left[1+\frac{1}{2{\left(1+\frac{2.5}{\kappa a\left\{1+2\exp\left(-\kappa a\right)\right\}}\right)}^{3}}\right]$$

where η is the viscosity of the solution, $U_{E}$ is the electrophoretic mobility, $\varepsilon_{r}$ is the relative permittivity of the solution, $\varepsilon_{0}$ is the permittivity of vacuum, $f\left(\kappa a\right)$ is the Henry’s function for a spherical colloidal particle of radius a, and κ is the reciprocal of the Debye length. In this study, κa was assumed to be sufficiently large; therefore, the $f\left(\kappa a\right)$ value was set to 1. The refractive index and absorption of the magnetite particles were set to 2.420 and 0.01, respectively. The principle for measuring the zeta potential is presented in detail [16]. After the measurement was finished, the solution in the cell was automatically transferred back to the sample container. The zeta potential measurements were automatically repeated from the second step by increasing the pH value from 9.0 to 10.0. No acid solution was used during the test. The magnetite nanoparticles remained thermodynamically stable during the zeta potential measurements in this study.

Furthermore, to compare the zeta potential of magnetite nanoparticles with and without the addition of NaCl, we prepared an additional sample solution by dispersing the magnetite nanoparticles with an average size of 5 nm in deionized water at a concentration of 25 mg/L. High-purity NaCl (99.99%) was added to the solution at a concentration of 6.6 mg/L. The pH of the solution was automatically titrated from 9.0 to 10.0 with diluted ETA. The test procedure was the same as that described above, and all observations were carried out at 25 °C.

### 2.2. Surface Zeta Potential Measurement

Particles in the suspension become charged and move under electrophoresis upon application of the electric field. Using these suspended particles as tracers, the surface zeta potential of metal can be estimated by measuring the electrophoretic mobility of the particles located at multiple distances from the metal surface.

**Figure 2:**
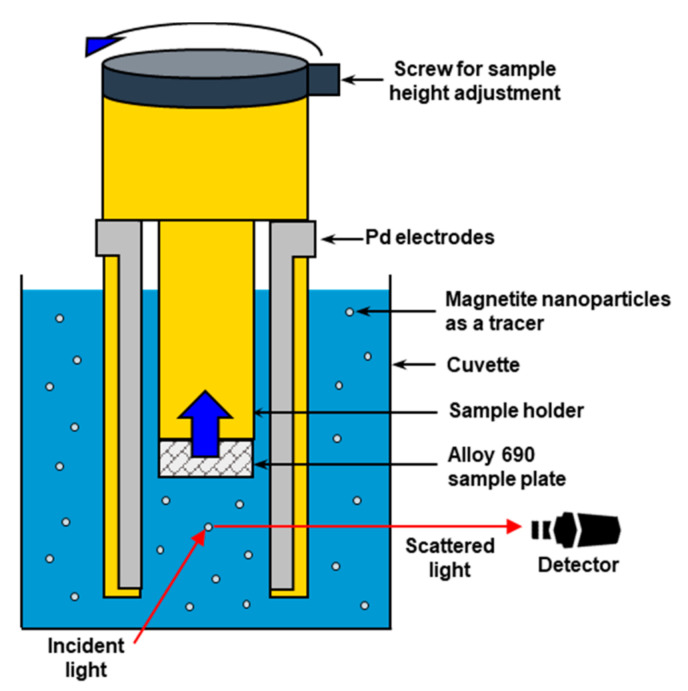
Schematic of a surface zeta potential measurement kit.

Figure 2 shows a schematic of the surface zeta potential measurement kit. Initially, we prepared rectangular samples of Alloy 690, which were not larger than 5 mm (length) × 4 mm (width) × 1 mm (thickness). To minimize the effect of surface conditions on the zeta potential [10], the sample surfaces were carefully ground to obtain a similar roughness. The sample was then attached to a sample holder and immersed in a solution in a cuvette, as shown in Figure 2. The solution was the same as that used to measure the zeta potential of magnetite nanoparticles as described in Section 2.1, and the pH was either 9.0 or 10.0. When an electric field is applied through a pair of Pd electrodes, the electrophoresis of tracer particles begins. We measured the apparent tracer electrophoretic mobility at four different distances from the sample surface by rotating a screw for adjusting the sample height. The surface zeta potential of Alloy 690 was then derived by the linear extrapolation method. All data were obtained at 25 °C; additional details have been described elsewhere [17].

Furthermore, surface zeta potential measurements using Alloy 690 were conducted to compare the results obtained with and without the addition of NaCl. The concentrations of magnetite particles and NaCl in the solution were the same as those of the solution used to measure the zeta potential, as described in Section 2.1. The pH was maintained at 9.5, with diluted ETA. The test procedure was the same as that mentioned above.

## 3. Results and Discussion

### 3.1. Effect of pH Control Agents

**Figure 3:**
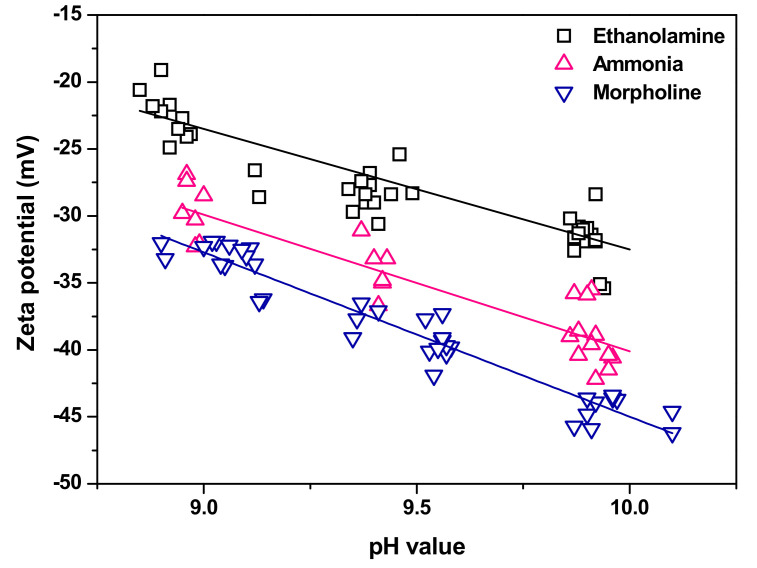
Zeta potentials of magnetite nanoparticles as a function of pH value, controlled with ethanolamine (ETA), ammonia, or morpholine at 25 °C.

Figure 3 shows the zeta potentials of magnetite nanoparticles as a function of pH value that is controlled with the addition of ETA, ammonia, or morpholine at 25 °C. According to previous studies, the zeta potential is dependent on the solution pH, and the relationship between zeta potential and pH is empirically expressed as [18,19]:

(3) $$\zeta =k\left(\mathrm{IEP}-\mathrm{pH}\right)$$

where k is a constant, and IEP is the isoelectric point, i.e., the pH value at which the zeta potential is equal to zero. In general, the zeta potential decreases as the pH increases. A decrease in the negative zeta potential to a more negative value implies greater repulsion to a negatively charged surface or particle [19]. The solid lines in Figure 3 show the linear regression fits of the measured zeta potentials in the solution of each pH agent based on Equation (3). The pH-dependent zeta potentials of magnetite nanoparticles at 25 °C can be expressed by the following empirical equations:

(4) $$\zeta_{E}=-9.0\times \mathrm{pH}+57.5\;(R^{2}=0.81)\;\zeta_{A}=-10.2\times \mathrm{pH}+61.9\;(R^{2}=0.81)\;\zeta_{M}=-12.3\times \mathrm{pH}+77.8\;(R^{2}=0.93)$$

where $\zeta_{E}$, $\zeta_{A}$, and $\zeta_{M}$ are for the solutions of ETA, ammonia, and morpholine, respectively. These equations are valid at pH values ranging from 9.0 to 10.0. In the future, if these equations are further upgraded by reflecting the effects of variables such as temperature, particle size, and shape, it is expected to be used in various ways as basic interfacial properties of magnetite particles suspended in secondary water. As shown in Figure 3, the magnetite nanoparticles were negatively charged under all conditions. Regardless of the pH agent, when the pH value increased from 9.0 to 10.0, the zeta potentials of the particles increased in the negative direction, which seems because the activity of hydroxyl groups (${\mathrm{OH}}^{-}$) increases with increasing pH value. This result implies that as the pH increases, the repulsive force between the particles increases, leading to an increase in dispersion stability and a decrease in the agglomeration. In addition, the zeta potential of the particles varied depending on the pH agent. At the same pH value, the absolute value of the zeta potential was the lowest when using ETA and the highest when using morpholine. According to a previous investigation [20], the electrical conductance of the magnetite column in the solutions increased in the order: ETA < ammonia < morpholine. This result implies that electrical properties of the particles vary depending on which solutions the particles are dispersed in; therefore, it is thought that the zeta potential of the particles appears to be high in the morpholine solution because the electrical conductance of the particles increases. Unfortunately, exactly what mechanism causes the zeta potential to be different depending on pH agent was not found in the literature. In fact, there are few studies comparable to our results with the exception of one previous investigation by Essi et al. [21]. They reported that the zeta potentials of magnetite were approximately −16 mV, −25 mV, and −36 mV when the pH was controlled at 9.2 with ETA, ammonia, and morpholine, respectively [21]. According to our results using Equation (4), the zeta potentials were calculated to be −25.3 mV, −32.0 mV, and −35.4 mV when the pH was 9.2 for the solutions of ETA, ammonia, and morpholine, respectively. The results for morpholine were almost similar, but the results for ETA and ammonia were evaluated to be greater in absolute values than those in the literature [21]. These differences may be caused by that the zeta potentials were measured by the streaming potential technique with a magnetite column made of 500 nm-sized magnetite powders in the previous study, whereas the zeta potentials were measured by the ELS technique with 5 nm-sized magnetite nanoparticles dispersed in the solutions in this study. On the other hand, based on the zeta potential results in this study, it is expected that the size of the magnetite particles should be the largest in the case of ETA, followed by ammonia and morpholine. This is because the lower the absolute value of zeta potential, the greater the tendency of particles to agglomerate.

**Figure 4:**
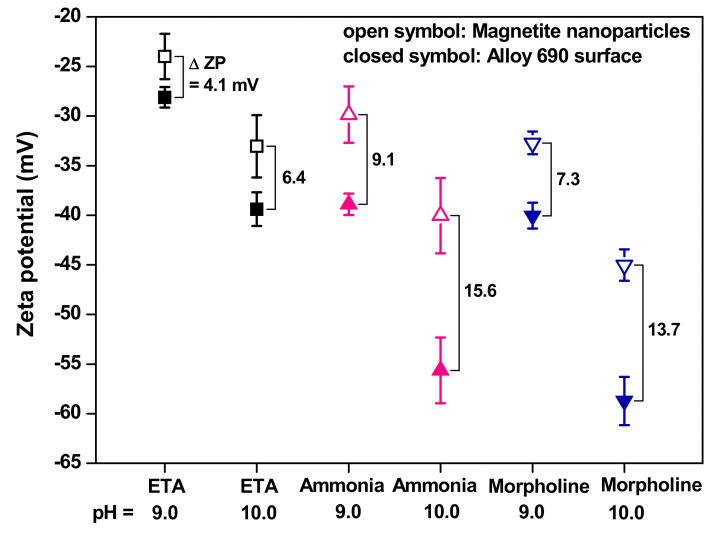
Relationship between the zeta potentials of magnetite nanoparticles and Alloy 690 surfaces at a pH of 9.0 and 10.0, depending on the pH agent at 25 °C.

Figure 4 shows the relationship between the zeta potentials of magnetite nanoparticles and Alloy 690 surfaces at pH values of 9.0 and 10.0, depending on the pH agent, at 25 °C. For all cases, the magnetite nanoparticles and Alloy 690 surfaces were negatively charged, thereby indicating that a repulsive force exists between them. As shown in Figure 4, the differences in the zeta potentials between the magnetite nanoparticles and Alloy 690 surfaces (∆ZP) could be calculated. When the pH increased from 9.0 to 10.0, ∆ZP increased regardless of the pH agent. Furthermore, ∆ZP indicates the electrostatic repulsive force between the particles and Alloy 690 surfaces; hence, the greater the ∆ZP, the more is the energy required to attach the particles to the surfaces. Accordingly, it is expected that the amount of magnetite deposition on the Alloy 690 surfaces will decrease as the pH increases from 9.0 to 10.0, regardless of the pH agent. In addition, at the same pH value, ∆ZP was the smallest in the ETA solution and the largest in the ammonia solution. Consequently, it is expected that the number of magnetite deposits will be the highest in ETA and the lowest in ammonia solution. However, the deposition behavior on the hot surface of operating SG tubes may not be fully explained by our results because the zeta potentials in this study were all measured at 25 °C. Nevertheless, considerable recent efforts have been made to improve this approach for a reliable description of the deposition behavior, along with the zeta potential measurement [9,10].

### 3.2. Effect of NaCl Addition

**Figure 5:**
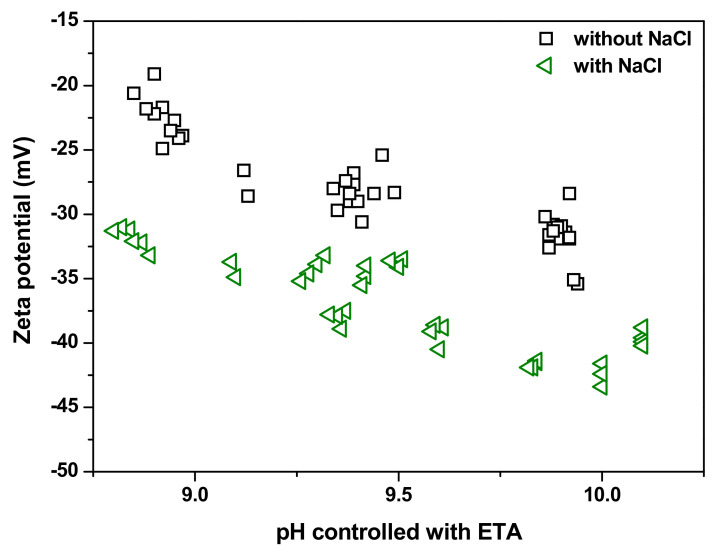
Zeta potentials of magnetite nanoparticles in solutions with and without NaCl addition as a function of pH value, controlled with ETA at 25 °C.

Figure 5 shows the effect of the addition of NaCl on the zeta potential of magnetite nanoparticles in solutions with pH values adjusted with ETA at 25 °C. As shown in the figure, even when NaCl was added, the magnetite particles were negatively charged, and the zeta potential increased further in the negative direction. Moreover, the zeta potential of the magnetite nanoparticles increased negatively as the pH increased from 9.0 to 10.0. Accordingly, the addition of NaCl increases the repulsive forces between the colloidal magnetite particles, thereby increasing the dispersion stability. Therefore, it is also expected that the agglomeration of the particles will be suppressed, which is also supported by the previous experimental results where the size of synthesized magnetite nanoparticles was found to decrease with increasing NaCl concentration [22].

**Figure 6:**
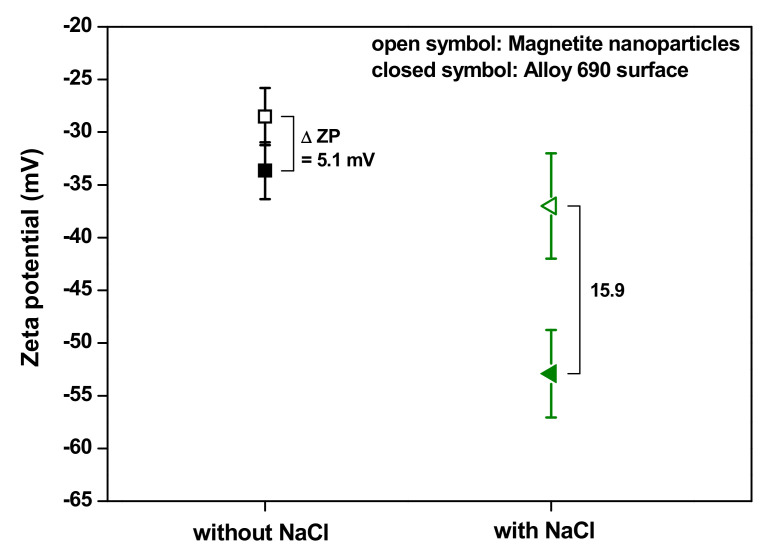
Relationship between the zeta potentials of magnetite nanoparticles and Alloy 690 surfaces at a pH of 9.5, controlled with ETA at 25 °C.

Figure 6 shows the relationship between the zeta potential of magnetite nanoparticles and the Alloy 690 surfaces at a pH of 9.5 controlled with ETA at 25 °C. The particles and surfaces were all negatively charged and the zeta potentials increased in the negative direction with the addition of NaCl. As shown in the figure, ∆ZP was calculated to be 5.1 mV and 15.9 mV without and with NaCl addition, respectively. These results indicate that the repulsive force between the particles and Alloy 690 surfaces increases with the addition of NaCl. Therefore, it is predicted that the deposition of magnetite particles on Alloy 690 surfaces requires more energy, leading to a decrease in the number of magnetite deposits owing to the presence of NaCl.

## 4. Conclusions

The zeta potentials of magnetite nanoparticles and Alloy 690 surfaces were measured at 25 °C. Based on the experimental results, the following conclusions were drawn:

1. The empirical formulas for the zeta potentials of magnetite nanoparticles derived in the pH range from 9.0 to 10.0 at 25 °C were as follows: $\zeta_{E}=-9.0\times \mathrm{pH}+57.5$ for ETA; $\zeta_{A}=-10.2\times \mathrm{pH}+61.9$ for ammonia; $\zeta_{M}=-12.3\times \mathrm{pH}+77.8$ for morpholine.
2. The zeta potentials of the magnetite nanoparticles increased in the negative direction as the pH increased from 9.0 to 10.0, regardless of the pH agent. This result indicates that as the pH increases, the repulsive force between the particles increases, thereby leading to an increase in the dispersion stability. At the same pH value, the absolute value of the zeta potential increased in the order: ETA < ammonia < morpholine, meaning that the dispersion stability of the particles also increases in the same order.
3. The difference in the zeta potentials between the magnetite nanoparticles and Alloy 690 surfaces increased with increasing pH from 9.0 to 10.0, regardless of the pH agent. At the same pH value, the difference was the largest in ammonia. From these results, it is expected that the amount of magnetite deposited on the Alloy 690 surfaces will decrease as the pH increases from 9.0 to 10.0, and will be the smallest in the ammonia.
4. The zeta potentials of magnetite nanoparticles increased in the negative direction by the addition of NaCl. In addition, the difference in the zeta potentials between the magnetite nanoparticles and Alloy 690 surfaces increased by approximately three times by the addition of NaCl. These results suggest that NaCl plays a role in increasing the repulsive force between the particles and Alloy 690 surfaces.

## Figures and Tables

**Table 1:** Basic properties of three pH agents used in this study.

pH Control Agents	Chemical Formula	Density (g/cm^3^)	Boiling Point (°C)	Molar Mass (g/mol)
Ammonia solution, 30%	NH_3_+H_2_O	0.89	36	17.0
Morpholine	C_4_H_9_NO	1.01	129	87.1
Ethanolamine (ETA)	C_2_H_7_NO	1.01	170	61.1
